# Educational Leader Reports of Statewide Change in Conditions for SEL Implementation over 1 Year of CalHOPE Student Support

**DOI:** 10.1007/s11121-025-01866-z

**Published:** 2026-01-08

**Authors:** Ashley N. Metzger, Justin D. Caouette, Patrick M. Robinson-Link, Jax Braun, CalHOPE Research Committee, Valerie B. Shapiro

**Affiliations:** 1 University of California, 120 Haviland Hall, Berkeley, CA 94720-7400 USA; 2 University of Washington, Seattle, WA USA

**Keywords:** Social and emotional learning (SEL), Implementation support, Statewide prevention infrastructure, Capacity building

## Abstract

This study examines changes in the conditions for social and emotional learning (SEL) implementation in California over the 2023–2024 school year, following approximately 3.5 years of CalHOPE Student Support. Grounded in the SHIFT-SEL model, CalHOPE aims to build statewide SEL infrastructure through a nested implementation support system, with County Offices of Education (COEs) positioned as key intermediaries. Educational leaders (507 in Fall 2023 and 386 in Spring 2024) reported on their well-being, workplace climate, and SEL implementation conditions. This study builds on prior work by including county, district, and school site SEL leaders in the analysis, enabling a broader examination of change across multiple levels of the education system. Cross-sectional comparisons showed that COE leaders reported more favorable conditions than district and school leaders, particularly in the extent of the supports they provided. Longitudinal analyses revealed small but significant improvements among district/school leaders in well-being, implementation supports, leadership capacities, and SEL structures and routines, while conditions at the COE level remained favorable. These findings suggest progress in infrastructure for SEL implementation support statewide and illustrate the potential of regional intermediaries for scaling prevention practice.

**Supplementary Information:**

The online version contains supplementary material available at 10.1007/s11121-025-01866-z.

Preventive interventions, such as school-based social and emotional learning (SEL) programs, have been demonstrated to have positive effects on the life trajectories of young people in the academic, social, emotional, and behavioral realms (Durlak et al., 2022). Calls to build infrastructure to support the wide-scale implementation of preventive interventions often envision local, regional, or statewide “backbone organizations” that coordinate multi-sector engagement (Shapiro & Bender, 2018) and provide implementation support (e.g., training, tools, technical assistance, and continuous improvement monitoring) to facilitate the high-quality delivery of preventative interventions in direct service settings (Brown et al., 2015; Wandersman et al., 2012). Creating comprehensive implementation support systems requires the development and testing of new systemic intervention frameworks that leverage multi-stakeholder partnerships and build workforce capacity (Fagan et al., 2019; Stanley et al., 2024), such as those seen within preventive interventions (Hawkins et al., 2002; Shapiro et al., 2013; Spoth et al., 2004; Wandersman et al., 2008). This paper explores the extent to which a new education system framework, put into practice over time, can engage partners, provide supports, and build capacity for advancing the structures and routines of SEL implementation.

## County Offices of Education as SEL Implementation Leaders

While classrooms, schools, and district settings have been studied with regard to SEL implementation (CASEL, 2020; Durlak et al., 2015; Kendziora & Yoder, 2016; Mart et al., 2015; Osher et al., 2016), educational service agencies (ESAs) have not been systematically studied. ESAs (e.g., Boards of Cooperative Educational Services, Educational Service Agencies/Districts, Intermediate Units, Regional Educational Service Centers) are “regional public multiservice agencies authorized by State law to develop, manage, and provide services or programs to local educational agencies” (20 USC § 1401(5) (2006)). In California, educational service agencies are referred to as County Offices of Education (COEs). A COE provides technical assistance, regional support activities, monitoring, and oversight related to academic environments and fiscal stability, and, at times, provides academic support and technical assistance to districts and schools within their county (California County Superintendents, 2024). COEs (and other ESAs) can support SEL implementation at scale by virtue of sitting upstream of more local entities . COEs may be able to promote *systemic SEL*—an approach to delivering SEL that engages each layer and component of a complex educational system to support, integrate, and sustain SEL (Elias et al., 1997; Shapiro et al., 2024). A California Department of Education (CDE) workgroup formalized recommendations to “design and implement a comprehensive capacity building system” to implement systemic SEL statewide (California Social and Emotional Learning State Team [CSELST], 2019), which began with building the capacity of the COEs (CSELEST, 2021). As argued, “County Offices of Education (COEs) play a central role within the State System of Support as the primary provider of professional development and technical assistance to districts… COEs serve as the most consistent and reliable conduit of information, initiatives, and practice improvement to districts across California.” (p. 8). At the time this recommendation was made to the State Superintendent of Public Instruction, there was no explicit theory of change for *how* to build the capacity of COEs to fulfill this role, nor a dedicated funding source for actualizing this vision.

## CalHOPE Student Support

CalHOPE Student Support (henceforth: CalHOPE) began as an emergency response to the COVID-19 pandemic, meeting the “social and emotional strains on leading, teaching, and learning” with an opportunity to “bring educational leaders into connection, capacity building, and collaboration.” (Shapiro, et al., 2024, p.3). With funding from the California Department of Healthcare Services, CalHOPE took up the aforementioned recommendations to build an adults-go-first prevention infrastructure to support the delivery of SEL in schools. In doing so, the CalHOPE State Planning Team synthesized and translated research evidence to create professional development resources for practice (see Metzger et al., 2024) for collective sense-making in a monthly statewide Community of Practice (CP) of COE representatives (see Eldeeb et al., 2025). The State Planning Team became a *secondary support system* (Lamont et al., 2024), acting to build the capacity of COEs to provide implementation support to others. As participating COE leaders developed their capacity, they then hosted regional CPs for local district and school site leaders. Thus, CalHOPE Student Support used progressive, nested communities of practice (Bohnenkamp et al., 2024), supplemented by customized technical assistance offered by COEs to districts and schools, to advance SEL delivery statewide.

## SHIFTing SEL Implementation

CalHOPE used *SHIFT-SEL* as a conceptual model to guide practice toward a more Systemic and Humanizing Implementation Focused on Transformation (Shapiro et al., In Press; see Figure SA). The model focuses on improving outcomes for young people *and* adults in the education sector in the domains of *well-being*, *engagement*, and *performance*. For adults, SHIFT contends that workplace *climate*—the persistent patterns of the setting that convey care and respect and create norms and expectations that transcend daily experiences—is associated with adult well-being and their successful engagement in and performance of activities associated with multi-systemic SEL implementation. These activities, conceptualized as *levers of transformation*, are postulated as systemic mechanisms of change. The first lever is *partnerships—*the trusting, equity-pursuing, and mutually beneficial collaborations between researchers and practitioners, spanning sectors and regions, between levels and divisions of the education system, with families, and adults allied to students that enable support for SEL implementation (Shapiro et al., 2024). The second lever is *support*—the funding, tools, training, coaching, and feedback systems that grow adult capacities for SEL implementation. The third lever is *capacity*—the mindsets, knowledge, skills, and confidence that adults require in order to change what they feel, think, and do. The final lever is the *structures and routines* of systemic SEL implementation (i.e., forming an SEL leadership team, creating a shared vision, conducting needs assessments, creating plans and goals, providing instruction or programs, developing a continuum of supports, and enacting data-based reflection and improvement). SHIFT-SEL also acknowledges the omnipresence of racism in the U.S. education system and therefore insists on the interrogation of equity in all model constructs. For more background on these constructs, please see Metzger and colleagues (2025a). Rather than concentrating on individuals within schools, or on individual school sites, SHIFT-SEL centers the larger education system—which research suggests is pivotal for sustainable change (Coburn et al., 2010; Daly & Finnigan, 2010, 2012; Fullan et al., 2009). Central offices have been under-conceptualized and under-studied in the literature, despite evidence that central office participation matters for change efforts (Burch & Spillane, 2004; Chrispeels et al., 2008; Daly & Finnigan, 2009; Finnigan & Daly, 2012; Honig, 2003; Honig & Copland, 2008; Johnson & Chrispeels, 2010). Successful systems change efforts rely on a collaborative cross-level approach (Burch & Spillane, 2004), and perceptions of authentic leadership and trust (Agote et al., 2016). SHIFT-SEL aims to describe the collaborative cross-level activities that become the mechanisms of systems change.

## Observations of SHIFTs in SEL Implementation

CalHOPE CPs began in January 2021. By August, nearly 6,000 educators had participated (Shapiro et al., 2024). Preliminary work revealed shifts in thinking about SEL “from skills to systems” among participants (Eldeeb et al., 2025), and a larger increase in the uptake of some evidence-informed SEL practices among California educators relative to the national average (Shapiro et al., 2024). In the Spring of 2022, COEs selected focal sites (i.e., three to eight schools upon which to focus supports to advance their SEL efforts) with whom to use their newly acquired capacities to provide implementation support. Ninety-five percent of counties articulated specific goals for providing implementation support, a majority of which focused on building regional infrastructure (e.g., developing district SEL plans; 64%) and strengthening adult SEL (e.g., engaging in professional learning; 53%; Metzger et al., 2025b).

By the summer of 2022, 72% of respondents to a survey of county leaders (representing 93% of California counties) said that there was a county-level SEL leadership team assembled in their region, and 65% of respondents reported that SEL was embedded in their COE’s strategic plan (Jones et al., Under Review). Approximately half (53%) of these respondents reported that their COE had a written plan with specific goals and strategies for providing SEL implementation support, with far fewer (28%) reporting a data system to benchmark progress against their implementation goals. Therefore, CalHOPE committed to the use of a data system aligned to the SHIFT model to support educational leaders in reflecting upon and continuously improving their work to advance multi-systemic SEL statewide: the Berkeley Assessment for Social and Emotional Learning (BASEL; Shapiro et al., 2022). In the summer of 2023, after 35 months of CalHOPE, county office leaders initially responded to the BASEL. They reported the strong presence of the levers of transformation, favorable workplace climate, and well-being (Metzger et al., 2025a). It remains unclear, however, the extent to which leaders in educational settings outside of county offices (e.g., districts, schools) are also experiencing favorable conditions for SEL implementation and the extent to which these conditions are improving over time.

## Current Study

The current study builds on prior work by including district- and school-level SEL leaders (in addition to COE leaders, who were included in prior work) in an exploration of the conditions of leading for SEL implementation, at one point in the typical school year, as well as extended prior analyses over time. We focus on two research questions (RQs).

RQ1. *To what extent do the well-being, climate, and levers of transformation differ among people providing SEL leadership at different levels of the education system?* We anticipate higher mean scores in well-being, workplace climate, and the levers of transformation among county-level leaders relative to district and school leaders. In a general context of high educator stress and burnout (Doan et al., 2022; Farley & Chamberlain, 2021), we expect differences due to functional differences in SEL implementation roles (i.e., implementation support versus delivery), and temporal differences in intervention. COEs first began (starting in January 2021) receiving statewide team supports for building levers of transformation (Shapiro et al., 2024). Thus, it was expected that levers would initially be stronger for COE leaders compared with district and school leaders, who were indirect and temporarily delayed recipients of CalHOPE supports. According to the SHIFT-SEL model, greater climate and well-being were hypothesized to be associated with the strength of levers (Shapiro et al., In Press) and thus are also hypothesized to be higher among COE leaders relative to district and school leaders. As district/school leaders were systematically included in CalHOPE later (starting mid 2023), it was expected that these level differences would diminish over time.

RQ2. *To what extent do the well-being, climate, and levers of transformation change between Fall 2023 and Spring 2024?* We hypothesized that there would be a positive change observed from Fall 2023 to Spring 2024 in SEL conditions, since CalHOPE aimed to continue improving these conditions. SHIFT-SEL stipulates a causal ordering among levers (Shapiro et al., 2024; Shapiro et al., In Press), starting with the building of partnerships. Partnership activities may enhance collective resources (Chesbrough, 2017; Van Beers & Zand, 2014), establish ongoing trust (Lin, 1999), and facilitate improvement through shared visioning (Finnigan & Daly, 2012). Partnerships, in turn, create the conditions for implementation support to be provided and received. Partnership-building was an early area of emphasis in CalHOPE. Thus, improvement was anticipated here, as well as in downstream supports (received, provided) enabled by partnership-building (Metzger et al., 2025a). When SEL supports are leveraged, they are theorized to enhance the capacities of educational leaders to implement SEL. Here, capacities were expected to increase, given the supports provided to COEs, and eventually by COEs to districts/schools. Finally, collective capacities are thought to enable the final lever: the structures and routines for SEL implementation. These levers, collectively, are hypothesized to serve as conditions for the promotion of leader workplace climate and well-being (Shapiro et al., In Press). To account for anticipated roles and timeline differences among education leaders at different levels, changes in SEL conditions were assessed separately at the COE level and were combined at the district/school levels.

## Method

### Data Collection and Recruitment

Data were collected in Fall 2023 and Spring 2024, approximately 40 and 45 months after the CalHOPE kick-off event, respectively. Prospective participants were sourced from a CalHOPE “Contact Directory”—a list of COE leaders contributing to CalHOPE, which was initiated and maintained by the State Planning Team. This directory also included COE leaders (e.g., division heads, county office superintendents) who were not involved in the day-to-day work of CalHOPE, but asked to be copied on communications by nature of their role overseeing employees advancing SEL. The second source of prospective county-level participants was prior survey respondents. This dual strategy created a broad, inclusive roster of prospective county-level participants.

Prospective district and school-level participants were derived through a referral system. Individuals listed in the contact directory were asked to identify district and school site leaders contributing to CalHOPE in their regions via a Qualtrics referral form. The referral form was pushed for approximately one month through CP meetings and emails. Additionally, county leaders were asked to forward the request to collaborating school and district leaders in their region. The contact directory and referral forms were continuously updated (more consistently when somebody needed to be added than when somebody should have been removed). Although email addresses were mostly usable, 9 and 118 emails bounced (i.e., did not reach the intended recipient) in the Fall and Spring respectively, including leaders with auto-response messages that indicated they were not performing their anticipated role during the entire data collection period (e.g., parental leave, shift in responsibilities, left organization). Ultimately, 1311 and 1240 personalized survey invitations were distributed in the Fall and Spring, respectively.

### Sample

Initial survey questions were used to filter the sample to educational leaders who responded affirmatively to at least one of two screening questions: “Are you in a role at your COE/district/school where you support social and emotional learning?” and “Are you on an SEL Leadership Team?” From across the state of California, 676 and 514 educational leaders in Fall 2023 and Spring 2024, respectively, responded. Of these, 657 (97.2%, Fall 2023) and 496 (96.5%, Spring 2024) met the screening criteria and were administered the full survey. A practice brief utilizing these data can be found online (Metzger et al., 2025c).

The analysis sample used here comprises the subsample of respondents who were administered the full survey *and* consented to have their data, initially collected for practice purposes, used for research. Consent was indeterminate for 81 (Fall) and 61 (Spring) respondents due to non-completion of consent-for-research items on the primarily-for-practice-purposes survey; these cases were excluded from analyses. The research-consenting sample (henceforth, participants) included 507 participants in Fall 2023 (88.0% consent rate; 38.7% of all contacted leaders) and 386 participants in Spring 2024 (88.7% consent rate; 31.1% of all contacted leaders). Confirmed with Chi-square tests, consenters were more likely than non-consenters to be non-Hispanic white (*p* <.05); there were no systematic differences by consent status on primary role in the education system (e.g., administration, instruction), frequency of student interaction, years of experience in current role, or gender. There were 357 screened-eligible participants who provided data at both time points, of which 257 consented (50.7% and 66.6% of Fall and Spring consenting samples, respectively). Participants represented 53 counties in Fall 2023 and 55 counties in Spring 2024 (out of 58 counties in California).

In the Fall 2023 sample, 79.6% were female. Participants reported on non-mutually exclusive categories for race and ethnicity: 3.2% were American Indian/Native Alaskan, 5.2% Asian/Asian American, 3.2% Black/African American, 18.9% Hispanic/Latinx, 0.8% Middle Eastern/North African/Arab American, 1.2% Native Hawaiian/Pacific Islander, 71.0% White/European American, and 5.0% Other. Among those leaders who reported having an SEL leadership team at their worksite (83.2% in Fall 2023 and 84.2% in Spring 2024), 92.7% of Fall and 95.1% of Spring participants identified as an SEL leadership team member. Observed characteristics were not significantly different across time points (Table S1).

The current study treats COE and district/school work settings as two discrete levels with independent samples. In practice, some educational leaders across the state serve in dual roles (e.g., a rural county leader may also be a school principal; an urban district leader of the only district in a county may therefore also be the county leadership). In this study, leaders were asked to describe their *primary* work by indicating whether their job is best described as situated within the (a) county office, or otherwise serving students across a whole county, (b) district office, or otherwise serving students across a whole district, or (c) school site, or otherwise serving students within a single school. In Fall 2023, participants were distributed across levels of the education system as follows: 23.7% county, 16.4% district, and 60.0% school. In Spring 2024, the distribution was: 33.2% county, 11.7% district, and 55.2% school. There was a significantly higher proportion of county-level participants in Spring 2024 (*χ*2 = 9.96, *p* =.002). Participants were categorized into two groups: COE leaders and district/school leaders. When comparing demographics across subsamples, there was a higher proportion of leaders working in districts/schools who had at least 6 years of experience in their current role as compared to leaders working at COEs in Spring 2024 (*χ*2 = 12.73, *p* <.001). No other sample characteristic differed by work setting at either time point (see Table S2 for full demographic information).

### Measures

The *Leader Voice* version of the BASEL (BASEL-LV, Shapiro et al., 2022) collects leader perceptions of their well-being, their workplace climate, and the presence of levers of transformation in their county, district, or school, to help facilitate needs assessment, prioritization, planning, and progress monitoring for SEL implementation. Participants respond to questions programmed in Qualtrics along a 4-point Likert scale (4: “YES!” means definitely true for you, 3: “yes” means mostly true for you; 2: “no” means mostly not true for you; 1: “NO!” means definitely not true for you). This scaling technique has been validated and used in multiple studies over the past 40 years (Social Development Research Group, 2005–2019). Some questions vary slightly based on the participant’s work setting (e.g., county: “Student voices guide our […county’s/district’s/school’s…] SEL implementation.” For each construct, the summary score is the mean of the scale or index items.

*Well-being* is conceptualized as two separate sub-constructs: *positive emotional experiences* and *resources for coping*. The positive emotional experiences (8 items) scale asks respondents to indicate positive affect over a two-week period (e.g., *proud*,* strong*, or* determined; grateful*,* thankful*, or *appreciative*; Cronbach’s *α* =.74). Two items indicating worry and sadness were reverse-coded. On the resources for coping index (5 items), respondents indicate their personal resources for coping with work-related stressors (e.g., “Even when things are hard, I remember why I work in education”) and general stressors (e.g., “I have skills to deal with difficult people and situations”). *Workplace climate* is conceptualized as three sub-constructs: *safety and connection* (3 items; e.g., “There is a collaborative work culture”; *α* =.83), *opportunities for leadership* (3 items; e.g., “My contributions are valued and respected”; *α* =.81), and experiences with culturally and linguistically responsive environments (3 items; e.g., “People consider the impact of race and racism on experiences in school”; *α* =.84).

The BASEL-LV also measures the four *levers of transformation*: *partnerships*, *supports* (*received* and *provided*), *capacities*, and *structures and routines for SEL implementation*. Each lever is measured using a separate scale, with scale scores being the mean of the items. An index measuring *partnership activities* indicates the types of activities that educational leaders engage in with existing teams, community members, and students (3 items; e.g., “Our COE collaborates across teams to advance SEL implementation”).

*Supports* are measured with two scales. A *supports received* scale (23 items; “Over the past 6 months, the CalHOPE Statewide team has provided opportunities for me to build my knowledge as an SEL leader”) refers to the supports that educational leaders received within the past 6 months to build capacity for SEL implementation. Although the types of supports reported on are identical for all leaders, the source of support differs by level. COE respondents report on supports received from the CalHOPE statewide team, while district respondents report on supports received from their COE, and school respondents report on supports received from their district and COE. The *supports provided* scale (15 items; e.g., “Over the past 6 months, our school SEL leaders have provided school staff with coaching (e.g., consultation, mentorship, feedback) for the practice of SEL”) refers to the supports that educational leaders provided within the past 6 months to build local capacity for SEL implementation. Again, here, the types of supports are identical for all leaders, but COE respondents report on supports provided to districts and schools, while district and school respondents report only on supports provided to school staff. Specific domains of support within these scales include funding, resources, training, tools, coaching, and feedback loops/systems. In earlier work, these domains of the support scale were indicated by items reflecting the practice of SEL, continuous improvement of SEL implementation (e.g., support for utilization of SEL data), and culturally and linguistically responsive practices that center and affirm student identities. Due to the high correlations observed between items assessing support for *SEL practice* and for *continuous improvement of SEL implementation*, and the desire for a brief tool, only continuous improvement items were retained (17/23 supports received, *α* =.93; 11/15 supports provided, *α* =.92).

A *capacities* scale (15 items) measures educational leaders’ perceptions of their own capacities for SEL implementation. It includes specific domains for *mindsets* (5 items; e.g., “I believe SEL is very important for student engagement”; *α* =.91), *knowledge* (3 items; e.g., “I am able to answer questions from people in my region about the practice of SEL”; *α* =.86), *skills* (6 items; e.g., “I have the skills to remove obstacles to SELimplementation”; *α* =.85), and sense of *efficacy* (1 item; “As a leader, I have confidence that I can support SEL implementation”). Psychometric analyses have revealed that the domains of the capacities scale demonstrated internal consistency, test–retest reliability, construct validity, temporal invariance, and factor structure invariance across COE and district/school level leaders (Caouette et al., 2025).

A scale for *structures and routines for SEL implementation* (18 items; *α* =.90) measures the presence of structures and routines needed for systemic SEL. It includes specific domains for SEL Leadership Team effectiveness (e.g., “Our SEL Leadership Team is effective at supporting SEL implementation”), having a shared vision (e.g., “Our district’s shared vision guides SEL implementation”), conducting needs assessments (e.g., “Our school has identified areas for SEL improvement based on a comprehensive needs assessment”), implementing plans (“In the past 6 months, our COE has used data to plan for SEL improvement in my region”), integrating SEL into programs/operations/instruction (e.g., “Our district office integrates SEL practices into our district’s daily activities and routines”), and data-based decision making for continuous improvement (e.g., “In the past6 months, our school has reviewed data to reflect upon SEL improvement in my school”).

### Data Analysis

Missingness on all key variables was small (0–2%), with data assumed to be missing at random (MAR). Missing data were handled with full information maximum likelihood (FIML) estimation on the outcome variable, with listwise deletion of a small number of cases with missing data on predictor variables. General linear models with mixed effects were run to test for differences in statewide mean-level well-being, work climate, and levers of transformation: (RQ1) between COE and district/school settings, and (RQ2) between Fall 2023 and Spring 2024. In all models for RQ1, the educational leader setting was the key predictor (coded as District/School = 0, COE = 1). For RQ2, timepoint was entered as the key predictor (coded as Fall 2023 = 0, Spring 2024 = 1). Analyses controlled for participant demographics, which were binarized for interpretability of the regression coefficients: 6 + years of teaching experience, female gender, and non-Hispanic white race/ethnicity. Measures of effect size were calculated as partial eta squared (η^2^) estimates. Effect sizes between.01 and.05 are considered small,.06–.13 medium, and.14 or above large (Richardson, 2011). Small effect sizes are most common in education research and were anticipated here, given the systemic nature of the intervention, large variability in work experiences, and the wide variety of school settings within the sample. Statistical significance of difference estimates was determined via two-tailed *p*-values, with a false discovery rate (FDR) correction implemented to account for the large number of tests.

For RQ1, analyses of level differences by setting—COE vs. district/school—were carried out separately in the Fall 2023 and Spring 2024 samples. The choice to combine district and school levels was supported by similar levels of SEL conditions reported by participants at these levels in Fall 2023. These models accounted for full nesting of participants within counties, since all participants were nested within counties. For RQ2, to account for the different nesting structures for participants within COE and district/school settings, analyses were conducted separately across these two settings. All models that were conducted with the COE sample controlled for clustering of individual participants within the same counties, while for the district/school sample only, models also controlled for clustering of individual participants within the same districts, which were fully nested within counties. For RQ2, a conditional clustering term was added to account for repeated measures among the subsample who completed the survey at both time points. As a sensitivity check for measuring change—and to rule out potential systematic influences of participants who dropped out of or had later entry into the dataset for reasons related to SEL constructs—analyses for RQ1 and RQ2 were repeated including only the subsamples of participants who completed the survey at both waves and remained in their primary work settings.

## Results

### Level Differences (RQ1)

See Table 1 for full details, including—for each timepoint and SEL condition—means and standard deviations within each educational setting, and key parameter estimates from regression analyses of level differences (i.e., beta coefficients, confidence intervals, raw and FDR-corrected *p*-values, ICCs). Across time points, county-level ICC values were small (range 0.00–0.15), indicating that county-level differences were small relative to individual-level differences. In Fall 2023, COE participants reported higher mean levels than district/school participants on all SEL conditions. Effect sizes were *large* for supports provided (η^2^ = 0.14), *small* for work climate—safety and connection, work climate—opportunities for leadership, partnership activities, supports received, all four measures of capacities, structures and routines, positive emotional experiences, and coping resources (η^2^ = 0.01–0.05), and *negligible* for work climate—cultural responsiveness (η^2^ = 0.00). In Spring 2024, COE leaders continued to report higher mean levels relative to other leaders (aside from no differences on structures and routines and positive emotional experiences). Overall, the gaps across levels narrowed, as evidenced by smaller regression coefficients in this time point. Effect sizes were medium for supports provided (η^2^ = 0.11), small for partnership activities, supports received, three of the capacities (knowledge, skills, efficacy), and coping resources (η^2^ = 0.01–0.05), and negligible for all three measures of work climate, capacities—mindsets, structures and routines, and positive emotional experiences (η^2^ = 0.00).

**Table 1:** Descriptive and inferential statistics comparing SEL conditions statewide in COE and district/school settings

		Descriptive statistics				Regression analyses						
		COE		District/school								
Timepoint	SEL condition	*M*	*SD*	*M*	*SD*	*B*	95% CI	*p*-value	FDR corr p	Partial eta sq	Effect size strength	ICC county
	*N*	120		387								
Fall 2023	Work Climate: Safety and Connection	3.21	*0.58*	3.10	*0.62*	0.10	[− 0.03, 0.23]	0.123	0.133	0.01	Small	0.00
	Work Climate: Opportunities for Leadership	3.37	*0.55*	3.27	*0.53*	0.09	[− 0.02, 0.21]	0.096	0.113	0.01	Small	0.02
	Work Climate: Cultural Responsiveness	2.89	*0.62*	2.87	*0.60*	0.01	[− 0.11, 0.14]	0.880	0.880	0.00	Negligible	0.04
	Partnership Activities	3.03	*0.55*	2.84	*0.54*	0.18	[0.07, 0.29]	0.002	0.004	0.02	Small	0.09
	Supports Received	3.27	*0.43*	3.03	*0.52*	0.25	[0.14, 0.35]	0.000	0.000	0.04	Small	0.11
	Supports Provided	3.32	*0.47*	2.86	*0.54*	0.48	[0.37, 0.58]	0.000	0.000	0.14	Large	0.12
	Capacities—Mindsets	3.86	*0.32*	3.79	*0.37*	0.07	[− 0.00, 0.14]	0.065	0.085	0.01	Small	0.01
	Capacities—Knowledge	3.50	*0.58*	3.29	*0.57*	0.19	[0.07, 0.31]	0.001	0.003	0.02	Small	0.00
	Capacities—Skills	3.33	*0.44*	3.07	*0.48*	0.26	[0.16, 0.35]	0.000	0.000	0.05	Small	0.01
	Capacities—Efficacy	3.62	*0.52*	3.44	*0.56*	0.17	[0.06, 0.29]	0.003	0.006	0.02	Small	0.02
	Structures and Routines	2.92	*0.53*	2.92	*0.51*	0.10	[− 0.00, 0.20]	0.062	0.085	0.01	Small	0.08
	Wellbeing: Positive Emotional Experiences	3.28	*0.39*	3.17	*0.40*	0.10	[0.02, 0.18]	0.020	0.033	0.01	Small	0.00
	Wellbeing: Coping Resources	3.30	*0.37*	3.10	*0.41*	0.18	[0.10, 0.27]	0.000	0.000	0.04	Small	0.01
	*N*	128		258								
Spring 2024	Work Climate: Safety and Connection	3.20	*0.62*	3.13	*0.62*	0.04	[− 0.09, 0.18]	0.547	0.674	0.00	Negligible	0.15
	Work Climate: Opportunities for Leadership	3.37	*0.53*	3.32	*0.58*	0.04	[− 0.09, 0.16]	0.570	0.674	0.00	Negligible	0.05
	Work Climate: Cultural Responsiveness	3.00	*0.61*	2.92	*0.61*	0.07	[− 0.07, 0.20]	0.332	0.480	0.00	Negligible	0.06
	Partnership Activities	3.08	*0.60*	2.93	*0.61*	0.17	[0.03, 0.30]	0.014	0.061	0.02	Small	0.09
	Supports Received	3.28	*0.43*	3.16	*0.55*	0.13	[0.02, 0.24]	0.021	0.068	0.01	Small	0.11
	Supports Provided	3.35	*0.50*	2.96	*0.59*	0.42	[0.29, 0.54]	0.000	0.000	0.11	Medium	0.10
	Capacities—Mindsets	3.89	*0.26*	3.85	*0.30*	0.04	[− 0.02, 0.10]	0.211	0.343	0.00	Negligible	0.00
	Capacities—Knowledge	3.55	*0.52*	3.43	*0.56*	0.11	[− 0.01, 0.23]	0.078	0.169	0.01	Small	0.02
	Capacities—Skills	3.38	*0.42*	3.15	*0.47*	0.23	[0.13, 0.33]	0.000	0.000	0.05	Small	0.00
	Capacities—Efficacy	3.63	*0.50*	3.53	*0.54*	0.10	[− 0.02, 0.21]	0.103	0.191	0.01	Small	0.00
	Structures and Routines	3.04	*0.54*	3.05	*0.52*	− 0.01	[− 0.12, 0.10]	0.897	0.897	0.00	Negligible	0.11
	Wellbeing: Positive Emotional Experiences	3.25	*0.36*	3.26	*0.42*	− 0.02	[− 0.11, 0.07]	0.641	0.694	0.00	Negligible	0.06
	Wellbeing: Coping Resources	3.29	*0.39*	3.17	*0.44*	0.09	[0.00, 0.18]	0.052	0.135	0.01	Small	0.03

Parameter estimates in regression analyses refer to the association between educational setting (coded as district/school = 0, COE = 1) and SEL condition, controlling for individual respondent demographics (years of teaching experience, gender, and race), and full nesting of all respondents within counties

*COE* County Office of Education, *B* unstandardized beta coefficient, *CI* confidence interval, *FDR corr p* false discovery rate corrected *p*-value, *ICC* intraclass correlation

### Change Scores (RQ2)

See Table 2 for full details of key parameter estimates from regression analyses of change scores (i.e., beta coefficients, confidence intervals, raw and FDR-corrected *p*-values, ICCs). ICC values were small at the district/school (range 0.01–0.16) and county (range 0.00–0.14) levels, indicating that differences at the district/school and county levels were small relative to individual-level differences. As expected, given the large number of survey repeaters, within-person ICCs were large, indicating a general pattern of consistency over time in responses among survey repeaters. In the COE setting, levels of SEL conditions were slightly higher in Spring 2024 than in Fall 2023 (with the exception of work climate—safety and connection, and positive emotional experiences, with negligible negative change). Positive effect sizes were *small* for work climate—cultural responsiveness, partnership activities, supports provided, and two capacities (knowledge, skills) (η^2^ = 0.01–0.02), and *negligible* for work climate—opportunities for leadership, supports received, two capacities (mindsets, efficacy), structures and routines, and coping resources (η^2^ = 0.00). In districts/schools, levels were higher in Spring 2024 than in Fall 2023 for all SEL conditions. Effect sizes were *small* for supports received and provided, three capacities (knowledge, skills, efficacy), structures and routines, positive emotional experiences, and coping resources (η^2^ = 0.01–0.03), and *negligible* for all three work climate measures, partnership activities, and capacities—mindsets (η^2^ = 0.00).

**Table 2:** Inferential statistics for change in SEL conditions statewide

Educational setting	*SEL condition*	Regression analyses								
		*B*	95% CI	*p*-value	FDR corr p	Partial eta sq	Effect size strength	ICC within-person	ICC district	ICC county
	*N* = *120 (Fall 2023) and 128 (Spring 2024)*									
COE	Work Climate: Safety and Connection	− 0.02	[− 0.15, 0.12]	0.820	0.967	0.00	Negligible	0.06	(–)	0.20
	Work Climate: Opportunities for Leadership	0.00	[− 0.12, 0.13]	0.951	0.967	0.00	Negligible	0.07	(–)	0.13
	Work Climate: Cultural Responsiveness	0.11	[− 0.03, 0.25]	0.114	0.927	0.02	Small	0.18	(–)	0.09
	Partnership Activities	0.05	[− 0.07, 0.17]	0.404	0.927	0.01	Small	0.34	(–)	0.09
	Supports Received	0.02	[− 0.07, 0.12]	0.620	0.967	0.00	Negligible	0.31	(–)	0.05
	Supports Provided	0.05	[− 0.06, 0.16]	0.380	0.927	0.01	Small	0.16	(–)	0.14
	Capacities—Mindsets	0.03	[− 0.04, 0.10]	0.428	0.927	0.00	Negligible	0.00	(–)	0.08
	Capacities—Knowledge	0.07	[− 0.06, 0.19]	0.290	0.927	0.01	Small	0.20	(–)	0.06
	Capacities—Skills	0.06	[− 0.04, 0.15]	0.240	0.927	0.01	Small	0.35	(–)	0.04
	Capacities—Efficacy	0.02	[− 0.09, 0.14]	0.691	0.967	0.00	Negligible	0.19	(–)	0.06
	Structures and Routines	0.02	[− 0.08, 0.13]	0.668	0.967	0.00	Negligible	0.36	(–)	0.11
	Wellbeing: Positive Emotional Experiences	− 0.01	[− 0.08, 0.07]	0.865	0.967	0.00	Negligible	0.33	(–)	0.10
	Wellbeing: Coping Resources	0.00	[− 0.09, 0.09]	0.967	0.967	0.00	Negligible	0.04	(–)	0.08
	*N* = *387 (Fall 2023) and 258 (Spring 2024)*									
District and school	Work Climate: Safety and Connection	0.01	[− 0.08, 0.10]	0.793	0.793	0.00	Negligible	0.31	0.15	0.03
	Work Climate: Opportunities for Leadership	0.04	[− 0.04, 0.11]	0.381	0.413	0.00	Negligible	0.35	0.03	0.04
	Work Climate: Cultural Responsiveness	0.04	[− 0.05, 0.13]	0.358	0.413	0.00	Negligible	0.35	0.04	0.04
	Partnership Activities	0.06	[− 0.02, 0.14]	0.148	0.192	0.00	Negligible	0.23	0.16	0.02
	Supports Received	0.11	[0.04, 0.18]	0.004	0.017	0.02	Small	0.26	0.11	0.07
	Supports Provided	0.08	[0.00, 0.16]	0.040	0.065	0.01	Small	0.25	0.05	0.14
	Capacities—Mindsets	0.05	[− 0.01, 0.10]	0.104	0.150	0.00	Negligible	0.02	0.01	0.00
	Capacities—Knowledge	0.13	[0.04, 0.21]	0.004	0.017	0.02	Small	0.22	0.01	0.03
	Capacities—Skills	0.08	[0.01, 0.15]	0.024	0.050	0.01	Small	0.29	0.02	0.03
	Capacities—Efficacy	0.10	[0.01, 0.18]	0.027	0.050	0.01	Small	0.16	0.01	0.02
	Structures and Routines	0.13	[0.06, 0.20]	0.001	0.013	0.03	Small	0.31	0.12	0.05
	Wellbeing: Positive Emotional Experiences	0.08	[0.02, 0.14]	0.006	0.020	0.02	Small	0.35	0.04	0.01
	Wellbeing: Coping Resources	0.07	[0.01, 0.12]	0.023	0.050	0.01	Small	0.41	0.02	0.00

Parameter estimates in regression analyses refer to the association between time (coded as Fall 2023 = 0, Spring 2024 = 1) and SEL condition, controlling for individual respondent demographics (years of teaching experience, gender, and race), partial nesting of individual respondents within repeated measures, and full nesting of district and school respondents within districts, which were fully nested within counties

*COE* County Office of Education, *B* unstandardized beta coefficient, *CI* confidence interval, *FDR corr p* false discovery rate corrected *p*-value, *ICC* intraclass correlation

### Sensitivity Analyses

For both RQ1 and RQ2, sensitivity analyses that included only survey repeaters were highly similar to results from the full sample. Across all models, there were very small differences in regression coefficients (<.10) and effect size estimates (<.05) between sensitivity and full sample analyses; see Tables S3 and S4.

## Discussion

CalHOPE Student Support, guided by SHIFT-SEL, was developed to advance SEL implementation across California. This study found that educational leaders in California, across diverse educational settings (COE, district, school), reported favorable conditions for supporting SEL implementation approximately 3.5 years after CalHOPE initiation. This study involved participants who had responsibilities for SEL and likely had pre-existing capacities (particularly, mindsets) for leading SEL initiatives; results likely apply most directly to leaders in similar conditions. COE leaders generally reported more favorable conditions than district/school leaders, with the most pronounced differences observed in the amount of supports they provided (e.g., large effect size in Fall 2023). This result makes sense in the context of CalHOPE, where COE leaders were positioned to be an important *source* of implementation support for districts and schools. It is unclear whether this hierarchical structure explains other differences (e.g., wellbeing), or whether differences are more attributable to different roles and stressors.

The gaps reported in conditions for SEL implementation support between COE leaders and district/school leaders appeared to be narrowing over time, particularly in regard to capacities for SEL implementation and adult well-being. Favorable change—albeit with small effect sizes—was observed in reports of district/school leaders across the 2023–2024 academic year in positive emotional experiences and coping resources, the amount of supports received and provided, the level of capacities (knowledge, skills, and efficacy), and the presence of structures and routines for SEL implementation. These findings are consistent with expectations, given that direct CalHOPE investment at the district and school levels came after COE investment, with COE conditions already highly favorable by Fall 2023, and district and school improvements emerging in the data over the course of the 2023–2024 school year. Although consistent with expectations, this change should not be taken for granted. We located no prior studies in the peer-reviewed literature showing systemic change in leader conditions for SEL implementation.

Overall, the results largely align with our hypotheses, particularly for differences across levels and for growth among district/school leaders. The general conditions for SEL implementation statewide are favorable and improving. Across 2023–2024, there was a sustained high presence of SEL levers at the COE level and observed improvements across most indicators at the district/school level. These results held up in sensitivity analyses that included only survey repeaters, bolstering confidence that results are not attributable to response bias. Where we see ceiling effects and small differences across levels or time, results may be biased downward, reflecting the already strong degree of investment in SEL among these leaders at study onset. This potential downward bias makes the consistent positive changes observed at the district/school level even more notable.

Within this overall favorable profile, it is also noteworthy that this study did not detect meaningful changes at the COE level, which may reflect a ceiling effect following earlier gains. Concordantly, COE mean scores from Fall 2023 (this study’s baseline) were generally higher than in COE mean scores in Summer 2022 (see Metzger et al., 2025a; non-COE leaders were not surveyed at this time). Specific dimensions may also have faced ceiling effects, such as favorable mindsets for SEL. Consistent with national surveys, school site leaders report valuing SEL, believing it should be taught, and believing in its benefits (DePaoli et al., 2017). This was true in this study also, among leaders across educational settings.

No meaningful differences across levels or time were observed for work climate constructs. This pattern may reflect that specific aspects of work climate—safety/connection, voice/leadership, cultural responsiveness—were not meaningfully different across settings or shaped by CalHOPE, such that COE and district/school leaders did not experience them differently over time as other SEL conditions improved. Relatively lower means were reported among COE and district/school SEL leaders for experiences of cultural responsiveness and the structures and routines of SEL implementation. These two dimensions had the lowest scores for COEs at both time points, with means of less than 3.00 in Fall 2023, and with similarly lower scores in districts/schools. Interestingly, cultural responsiveness among COE leaders was consistent from Summer 2023 (Metzger et al., 2025a) to Fall 2023, and grew slightly, though not significantly, from Fall 2023 to Spring 2024. Ongoing monitoring may reveal a positive trend over time. As for Structures and Routines, it is not surprising that this dimension had the lowest mean score, considering it is theorized as coming temporally last in the SHIFT-SEL theory of change. It is also consistent with a national survey of school principals, which concluded that few schools fully meet systemic SEL implementation benchmarks (DePaoli et al., 2017). For example, this report indicated that less than half of principals have a schoolwide vision statement for SEL and “only about one-third of principals (35 percent) report that their school has developed a plan for teaching students social and emotional skills and is systematically implementing it schoolwide” (p. 20). It is possible that CalHOPE engagement accelerates progress against this national benchmark, and that, over time, we may see a positive trend here as well.

Change in the conditions for SEL implementation over time could reflect many aspects of the education system that are dynamic in nature. Patterns of change over time and across levels of the education system could reflect the sequence of the CalHOPE project roll-out, similar levels of improvement across all levels during CalHOPE that were layered on top of meaningful differences before CalHOPE, or the nature of the different SEL leadership contexts and roles at the different levels. Yet, findings could also provide some evidence that a centralized system of implementation support, as theorized by Wandersman and colleagues (2008), can facilitate not only the dissemination and implementation of innovations, but also a collaborative improvement-oriented approach to systemic SEL. Hwang and colleagues (2025) qualitatively analyzed regional CalHOPE plans, discovering an emergent characterization of COE orientations to implementation support as “push” (top-down) or “pull” (bottom-up) strategies. In future research, this may be explored as a moderator of successful system change. In a broader sense, further data collection and analysis may contribute to additional understanding of “why” and/or “how” the conditions for SEL implementation and leader well-being are improving over time.

## Limitations

The current study was based on a sample of educational leaders working within a single statewide educational system and indicating at least some involvement in the provision of SEL support. Thus, the study’s results may not generalize to other statewide K-12 systems or to educational leaders not working as part of CalHOPE. Nevertheless, the sample—which spans nearly every county in California—represents a diverse array of educational settings that are embedded within diverse social and cultural systems. Because the BASEL-LV asks general questions about SEL conditions rather than specific questions about implementation supports within the state of California or within the CalHOPE system, the findings here are likely to be transportable to other K-12 systems and even ripe for cross-state comparisons.

Another limitation of this study was the high likelihood of selection bias. Survey completion was pro-actively asserted as a condition of COE participation in CalHOPE, but this MOU term may or may not have been included in subcontracts between COEs and districts/schools, and was not tracked for compliance purposes. Participants were not offered individual compensation for their time completing the survey. It is possible that educational leaders who chose to provide survey data differed systematically from survey non-responders in relevant characteristics, such as being overly attentive to—or overly optimistic about—the favorable impact of CalHOPE. We were unable to triangulate survey responses with other data sources to pursue this line of inquiry.

We also acknowledge general limitations to reliability and validity. Some study participants had long-standing involvement with CalHOPE, and in the setting they were assessing, while others may have been relatively new at the time of survey administration. Participants were encouraged to complete the survey regardless. Further, this study does not enable us to directly test connections between any of the SHIFT model constructs and the success of CalHOPE’s implementation of SEL or any adult or student outcomes.

## Conclusion

This study provides evidence of shifts in conditions for the implementation of SEL in California during the 2023–2024 school year, following approximately 3.5 years of CalHOPE Student Support. Findings highlight both the progress and ongoing challenges of building sustainable and systemic SEL infrastructure statewide. While COE leaders consistently reported favorable conditions, district and school leaders demonstrated small but significant improvements in well-being, supports, capacities, and the structures and routines for SEL. These results underscore the value of positioning regional intermediaries as the anchors for scaling prevention practice and advancing systemwide SEL capacity. Continued investment in capacity-building for SEL among educational leaders across the system will be critical to sustaining momentum and driving long-term transformation in SEL implementation.

## Supplementary Information

Below is the link to the electronic supplementary material.

ESM 1 Supplementary Material 1 (PDF 57.6 KB)

ESM 2 Supplementary Material 2 (PDF 75.7 KB)

ESM 3 Supplementary Material 3 (PDF 72.3 KB)

ESM 4 Supplementary Material 4 (PDF 68.3 KB)

ESM 5 Supplementary Material 5 (DOCX 137 KB)

## Data Availability

Please contact the corresponding author for inquiries regarding data availability.
