# Supplementary material for: Educational Leader Reports of Statewide Change in Conditions for SEL Implementation over 1 Year of CalHOPE Student Support
Source: Prev Sci. 2026 Jan 8;26(8):1263–75. doi: 10.1007/s11121-025-01866-z (PMC12804232; doi:10.1007/s11121-025-01866-z)
Supplement: Supplementary file 1 — Supplementary Material 1 (PDF 57.6 KB) [file 11121_2025_1866_MOESM1_ESM.pdf]

Table S1. Sample Description

	Fall 2023	Spring 2024
Primary setting is COE	23.7%**a**	33.2%**a**
Primary role is instructor	34.1%	32.6%
COE	28.3%	28.1%
District/School	35.9%	34.9%
6+ years in current role	44.2%	39.8%
COE	37.5%	26.8%**b**
District/School	46.3%	46.3%**b**
Non-Hispanic white	71.0%	71.8%
COE	76.3%	75.2%
District/School	69.4%	70.2%
Female	79.6%	80.4%
COE	79.8%	82.5%
District/School	79.6%	79.4%

Note. Due to missing data, sample sizes within each timepoint and educational setting differ among demographic variables. COE = County Office of Education.

aPercentages differ significantly between Fall 2023 and Spring 2024 in a Chi square difference test ($p < .05$)

bPercentages differ significantly between COE and District/School samples in a Chi square difference test ($p < .05$)
