# Supplementary material for: Educational Leader Reports of Statewide Change in Conditions for SEL Implementation over 1 Year of CalHOPE Student Support
Source: Prev Sci. 2026 Jan 8;26(8):1263–75. doi: 10.1007/s11121-025-01866-z (PMC12804232; doi:10.1007/s11121-025-01866-z)
Supplement: Supplementary file 2 — Supplementary Material 2 (PDF 75.7 KB) [file 11121_2025_1866_MOESM2_ESM.pdf]

Table S2. Sample Description with Demographic Categories Disaggregated

	Fall 2023			Spring 2024		
Primary work setting	**COE**	**District**	**School**	**COE**	**District**	**School**
N	120	83	304	128	45	213
**Nesting Trends**						
Spring 2024 respondents repeated from Fall 2023	(--)	(--)	(--)	62.6%	63.0%	63.7%
Number of counties represented	50	35	48	51	27	49
Number of districts represented	(--)	57	106	(--)	38	102
Median (IQR) number of respondents in each county	3 [2, 4]	3 [2, 4]	12 [5, 15]	3 [2, 4]	2 [1, 3]	6 [5, 11]
Median (IQR) number of respondents in each district	(--)	2 [1, 2]	4 [2, 7]	(--)	1 [1, 2]	3 [2, 5]
**Sample Demographics**						
Role supports SEL implementation	100.0%	100.0%	99.3%	99.2%	100.0%	97.7%
Setting has an SEL Leadership Team on site	85.8%	77.1%	83.9%	86.7%	73.3%	85.0%
Respondent is on an SEL Leadership Team	87.4%	89.1%	95.7%	91.0%	97.0%	97.2%
Primary role in K-12 education						
Administration	50.0%	51.8%	28.6%	43.0%	57.8%	31.5%
Instruction	28.3%	10.8%	42.8%	28.1%	11.1%	39.9%
Student Wellbeing	19.2%	34.9%	25.7%	20.3%	26.7%	24.9%
None of these	2.5%	2.4%	3.0%	8.6%	4.4%	3.8%
Frequency of student interaction during work week						
Almost all of the time	4.2%	16.9%	72.7%	6.2%	13.3%	68.1%
Most of the time	5.0%	18.1%	16.8%	3.9%	11.1%	19.2%
Some of the time	7.5%	19.3%	9.2%	10.9%	24.4%	10.3%
Not a lot of the time	83.3%	45.8%	1.3%	78.9%	51.1%	2.3%
Years worked in current role						
0-1 year	17.5%	16.9%	11.8%	23.6%	15.6%	10.4%
2-5 years	45.0%	51.8%	37.8%	49.6%	53.3%	40.1%
6-10 years	23.3%	19.3%	20.4%	15.0%	20.0%	22.6%
11-15 years	10.0%	2.4%	9.9%	7.9%	6.7%	7.1%
16-20 years	3.3%	4.8%	6.6%	2.4%	0.0%	6.6%
More than 20 years	0.8%	4.8%	13.5%	1.6%	4.4%	13.2%
Race and ethnicity						

American Indian/Native Alaskan	2.5%	3.7%	3.3%	4.8%	2.3%	4.3%
Asian/Asian American	5.9%	6.1%	4.6%	4.0%	4.5%	4.7%
Black/African American	3.4%	4.9%	2.6%	6.4%	2.3%	4.7%
Hispanic/Latinx	14.4%	28.0%	18.2%	14.4%	18.2%	20.4%
Middle Eastern/North African/Arab American	0.0%	3.7%	0.3%	2.4%	0.0%	0.9%
Native Hawaiian/Pacific Islander	1.7%	1.2%	1.0%	0.8%	0.0%	2.4%
Non-Hispanic White/European American	76.3%	61.0%	71.6%	75.2%	77.3%	68.7%
Other	2.5%	6.1%	5.6%	1.6%	0.0%	7.1%
Gender						
Woman	79.8%	77.1%	80.3%	82.5%	73.3%	80.7%
Man	19.3%	20.5%	19.1%	15.9%	22.2%	17.9%
Transgender	0.8%	2.4%	0.0%	0.0%	2.2%	0.0%
Non-binary	0.0%	0.0%	0.3%	0.0%	0.0%	0.0%
Other	0.0%	0.0%	0.7%	1.6%	2.2%	1.4%
Prefer not to answer	0.0%	1.2%	0.0%	0.0%	0.0%	0.0%

Note. Due to missing data, sample sizes within each timepoint and educational setting differ among demographic variables.
COE = County Office of Education; IQR = interquartile range.
