# Supplementary material for: Educational Leader Reports of Statewide Change in Conditions for SEL Implementation over 1 Year of CalHOPE Student Support
Source: Prev Sci. 2026 Jan 8;26(8):1263–75. doi: 10.1007/s11121-025-01866-z (PMC12804232; doi:10.1007/s11121-025-01866-z)
Supplement: Supplementary file 3 — Supplementary Material 3 (PDF 72.3 KB) [file 11121_2025_1866_MOESM3_ESM.pdf]

**Table S3**. Inferential Statistics Comparing SEL Conditions Statewide in COE and District/School Settings: Survey Repeaters Only

| | | Regression Analyses | | | | | | |
|---|---|---|---|---|---|---|---|---|
| Timepoint | SEL Condition | *B* | 95% CI | p-value | FDR corr p | Partial eta sq | Effect Size Strength | ICC County |
| | *N = 82 (COE) and 171 (District/School)* | | | | | | | |
| | Work Climate: Safety and Connection | 0.09 | [-0.06, 0.25] | 0.244 | 0.286 | 0.01 | small | 0.00 |
| | Work Climate: Opportunities for Leadership | 0.15 | [0.01, 0.28] | 0.035 | 0.057 | 0.02 | small | 0.00 |
| | Work Climate: Cultural Responsiveness | 0.09 | [-0.07, 0.25] | 0.273 | 0.286 | 0.01 | small | 0.04 |
| | Partnership Activities | 0.18 | [0.04, 0.31] | 0.011 | 0.020 | 0.03 | small | 0.17 |
| | Supports Received | 0.20 | [0.08, 0.32] | 0.002 | 0.007 | 0.04 | small | 0.10 |
| | Supports Provided | 0.45 | [0.32, 0.58] | 0.000 | 0.000 | 0.16 | large | 0.11 |
| Fall 2023 | Capacities - Mindsets | 0.07 | [-0.01, 0.15] | 0.075 | 0.108 | 0.01 | small | 0.03 |
| | Capacities - Knowledge | 0.20 | [0.05, 0.35] | 0.011 | 0.020 | 0.03 | small | 0.00 |
| | Capacities - Skills | 0.27 | [0.14, 0.39] | 0.000 | 0.000 | 0.07 | medium | 0.02 |
| | Capacities - Efficacy | 0.21 | [0.07, 0.36] | 0.004 | 0.010 | 0.03 | small | 0.06 |
| | Structures and Routines | 0.07 | [-0.06, 0.20] | 0.286 | 0.286 | 0.01 | small | 0.13 |
| | Wellbeing: Positive Emotional Experiences | 0.09 | [-0.02, 0.19] | 0.117 | 0.152 | 0.00 | negligible | 0.00 |
| | Wellbeing: Coping Resources | 0.20 | [0.09, 0.30] | 0.000 | 0.000 | 0.05 | small | 0.05 |
| | *N = 82 (COE) and 171 (District/School)* | | | | | | | |
| | Work Climate: Safety and Connection | 0.13 | [-0.04, 0.29] | 0.131 | 0.213 | 0.01 | small | 0.09 |
| | Work Climate: Opportunities for Leadership | 0.08 | [-0.07, 0.23] | 0.317 | 0.412 | 0.00 | negligible | 0.03 |
| | Work Climate: Cultural Responsiveness | 0.11 | [-0.05, 0.28] | 0.172 | 0.248 | 0.01 | small | 0.03 |
| | Partnership Activities | 0.20 | [0.04, 0.37] | 0.015 | 0.049 | 0.03 | small | 0.14 |
| | Supports Received | 0.16 | [0.03, 0.30] | 0.022 | 0.049 | 0.02 | small | 0.10 |
| | Supports Provided | 0.46 | [0.31, 0.61] | 0.000 | 0.000 | 0.13 | medium | 0.10 |
| Spring 2024 | Capacities - Mindsets | 0.02 | [-0.06, 0.09] | 0.649 | 0.684 | 0.00 | negligible | 0.00 |
| | Capacities - Knowledge | 0.17 | [0.02, 0.31] | 0.023 | 0.049 | 0.02 | small | 0.00 |
| | Capacities - Skills | 0.29 | [0.17, 0.40] | 0.000 | 0.000 | 0.08 | medium | 0.02 |
| | Capacities - Efficacy | 0.16 | [0.01, 0.30] | 0.034 | 0.063 | 0.02 | small | 0.01 |
| | Structures and Routines | 0.04 | [-0.10, 0.18] | 0.561 | 0.663 | 0.00 | negligible | 0.11 |
| | Wellbeing: Positive Emotional Experiences | -0.03 | [-0.13, 0.09] | 0.684 | 0.684 | 0.00 | negligible | 0.05 |
| | Wellbeing: Coping Resources | 0.13 | [0.02, 0.24] | 0.022 | 0.049 | 0.02 | small | 0.02 |

*Note*. Parameter estimates in regression analyses refer to the association between educational setting (coded as District/School = 0, COE = 1) and SEL condition, controlling for individual respondent demographics (years of teaching experience, gender, and race), and full nesting of all respondents within counties. Four participants changed primary setting between COE and District/School from Fall 2023 to Spring 2024 and were excluded from analyses. *COE* = County Office of Education; *B* = unstandardized beta coefficient; *CI* = confidence interval; FDR corr p = false discovery rate corrected p-value; *ICC* = intraclass correlation
