# Supplementary material for: Educational Leader Reports of Statewide Change in Conditions for SEL Implementation over 1 Year of CalHOPE Student Support
Source: Prev Sci. 2026 Jan 8;26(8):1263–75. doi: 10.1007/s11121-025-01866-z (PMC12804232; doi:10.1007/s11121-025-01866-z)
Supplement: Supplementary file 4 — Supplementary Material 4 (PDF 68.3 KB) [file 11121_2025_1866_MOESM4_ESM.pdf]

Table S4. Inferential Statistics for Change in SEL Conditions Statewide: Survey Repeaters Only

Educational Setting	SEL Condition	*B*	95% CI	p-value	FDR corr p	Partial eta sq	Effect Size Strength	ICC Within-Person	ICC District	ICC County
		Regression Analyses								
	N = 82									
COE	Work Climate: Safety and Connection	-0.03	[-0.15, 0.09]	0.601	0.772	0.00	negligible	0.39	(--)	0.15
	Work Climate: Opportunities for Leadershir	-0.09	[-0.19, 0.01]	0.087	0.566	0.04	small	0.49	(--)	0.00
	Work Climate: Cultural Responsiveness	0.03	[-0.10, 0.16]	0.653	0.772	0.00	negligible	0.27	(--)	0.26
	Partnership Activities	0.05	[-0.06, 0.16]	0.389	0.772	0.01	small	0.51	(--)	0.14
	Supports Received	0.03	[-0.06, 0.11]	0.508	0.772	0.01	small	0.56	(--)	0.02
	Supports Provided	0.07	[-0.03, 0.16]	0.189	0.614	0.02	small	0.36	(--)	0.21
	Capacities - Mindsets	-0.01	[-0.08, 0.06]	0.792	0.811	0.00	negligible	0.14	(--)	0.10
	Capacities - Knowledge	0.08	[-0.03, 0.20]	0.162	0.614	0.03	small	0.34	(--)	0.17
	Capacities - Skills	0.08	[-0.00, 0.17]	0.065	0.566	0.04	small	0.57	(--)	0.00
	Capacities - Efficacy	-0.01	[-0.13, 0.11]	0.811	0.811	0.00	negligible	0.26	(--)	0.21
	Structures and Routines	0.03	[-0.06, 0.13]	0.520	0.772	0.01	small	0.48	(--)	0.22
	Wellbeing: Positive Emotional Experiences	-0.02	[-0.10, 0.06]	0.546	0.772	0.01	small	0.56	(--)	0.00
	Wellbeing: Coping Resources	0.03	[-0.06, 0.12]	0.501	0.772	0.01	small	0.20	(--)	0.14
	N = 171									
District & School	Work Climate: Safety and Connection	-0.08	[-0.15, -0.00]	0.047	0.176	0.02	small	0.61	0.00	0.08
	Work Climate: Opportunities for Leadershir	-0.05	[-0.12, 0.02]	0.184	0.299	0.01	small	0.63	0.00	0.00
	Work Climate: Cultural Responsiveness	0.00	[-0.08, 0.08]	0.946	0.946	0.00	negligible	0.60	0.04	0.00
	Partnership Activities	0.02	[-0.06, 0.09]	0.692	0.750	0.00	negligible	0.44	0.00	0.17
	Supports Received	0.01	[-0.01, 0.12]	0.096	0.188	0.02	small	0.56	1.23	0.09
	Supports Provided	0.03	[-0.05, 0.11]	0.412	0.536	0.00	negligible	0.48	0.00	0.07
	Capacities - Mindsets	0.00	[-0.03, 0.07]	0.495	0.585	0.00	negligible	0.32	0.06	0.00
	Capacities - Knowledge	0.10	[0.02, 0.18]	0.016	0.104	0.03	small	0.49	0.00	0.02
	Capacities - Skills	0.05	[-0.01, 0.12]	0.101	0.188	0.02	small	0.58	0.00	0.00
	Capacities - Efficacy	0.04	[-0.05, 0.14]	0.381	0.536	0.01	small	0.31	0.00	0.02
	Structures and Routines	0.06	[-0.01, 0.13]	0.074	0.188	0.02	small	0.58	0.00	0.06
	Wellbeing: Positive Emotional Experiences	0.06	[-0.00, 0.12]	0.054	0.176	0.02	small	0.57	0.01	0.00
	Wellbeing: Coping Resources	0.07	[0.01, 0.12]	0.014	0.104	0.04	small	0.67	3.96	0.01

Note. Parameter estimates in regression analyses refer to the association between time (coded as Fall 2023 = 0, Spring 2024 = 1) and SEL condition, controlling for individual respondent demographics (years of teaching experience, gender, and race), nesting of individual respondents within repeated measures, and full nesting of district and school respondents within districts, which were fully nested within counties. Four participants changed primary setting between COE and District/School from Fall 2023 to Spring 2024 and were excluded from analyses. *COE* = County Office of Education; *B* = unstandardized beta coefficient; *CI* = confidence interval; FDR corr p = false discovery rate corrected p-value; *ICC* = intraclass correlation
