# Supplementary material for: Educational Leader Reports of Statewide Change in Conditions for SEL Implementation over 1 Year of CalHOPE Student Support
Source: Prev Sci. 2026 Jan 8;26(8):1263–75. doi: 10.1007/s11121-025-01866-z (PMC12804232; doi:10.1007/s11121-025-01866-z)
Supplement: Supplementary file 5 — Supplementary Material 5 (DOCX 137 KB) [file 11121_2025_1866_MOESM5_ESM.docx]

**Figure SA**

*The SHIFT Model*


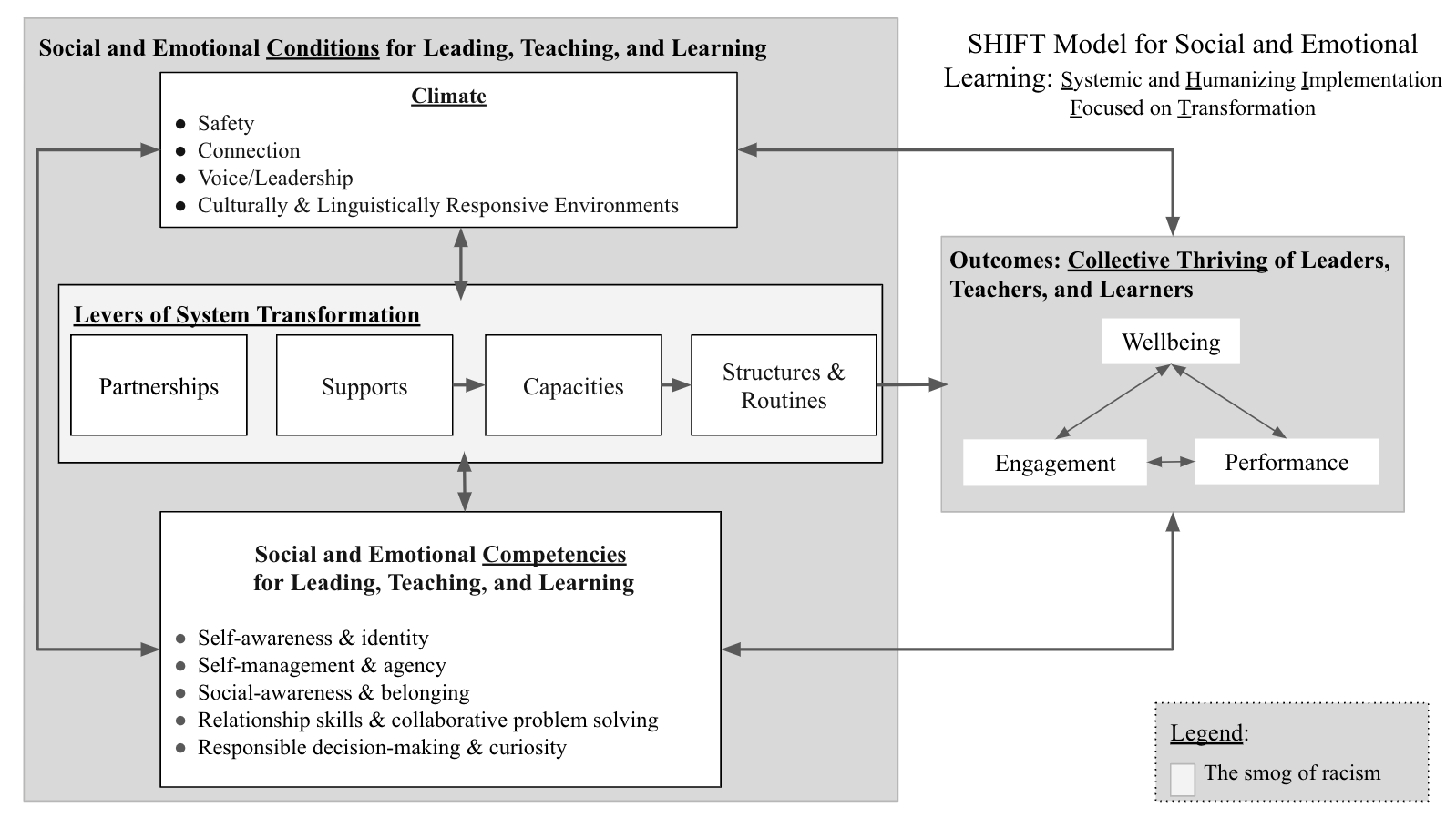


Note: Image copyright retained by the authors. Please inquire with the corresponding author for permission to copy or reproduce.
